# Screening of alginate lyase-excreting microorganisms from the surface of brown algae

**DOI:** 10.1186/s13568-017-0361-x

**Published:** 2017-04-04

**Authors:** Mingpeng Wang, Lei Chen, Zhaojie Zhang, Xuejiang Wang, Song Qin, Peisheng Yan

**Affiliations:** 1 School of Municipal and Environmental Engineering, Harbin Institute of Technology, Harbin, 150090 China; 2 Yantai Institute of Costal Zone Research Chinese Academy of Sciences, 17 Chunhui Road, Yantai, 264003 Shandong province China; 3 Department of Zoology and Physiology, University of Wyoming, Laramie, WY USA; 4 Worldfull Agricultural Science and Technology Co., Ltd, Yantai, 264000 China; 5 School of Marine Science and Technology, Harbin Institute of Technology at Weihai, West Culture Road 2, Weihai, 264209 Shandong Province China

**Keywords:** Alginate lyase, Gram’s iodine, Alginate degradation, Screening, Brown algae

## Abstract

Alginate lyase is a biocatalyst that degrades alginate to produce oligosaccharides, which have many bioactive functions and could be used as renewable biofuels. Here we report a simple and sensitive plate assay for screening alginate lyase-excreting microorganisms from brown algae. Brown algae *Laminaria japonica*, *Sargassum horneri* and *Sargassum siliquatrum* were cultured in sterile water. Bacteria growing on the surface of seaweeds were identified and their capacity of excreting alginate lyase was analyzed. A total of 196 strains were recovered from the three different algae samples and 12 different bacterial strains were identified capable of excreting alginate lyases. Sequence analysis of the 16S rRNA gene revealed that these alginate lyase-excreting strains belong to eight genera: *Paenibacillus* (4/12), *Bacillus* (2/12), *Leclercia* (1/12), *Isoptericola* (1/12), *Planomicrobium* (1/12), *Pseudomonas* (1/12), *Lysinibacillus* (1/12) and *Sphingomonas* (1/12). Further analysis showed that the LJ-3 strain (*Bacillus halosaccharovorans*) had the highest enzyme activity. To our best knowledge, this is the first report regarding alginate lyase-excreting strains in *Paenibacillus*, *Planomicrobium* and *Leclercia.* We believe that our method used in this study is relatively easy and reliable for large-scale screening of alginate lyase-excreting microorganisms.

**Electronic supplementary material:**

The online version of this article (doi:10.1186/s13568-017-0361-x) contains supplementary material, which is available to authorized users.

## Introduction

Alginate is an acidic linear polysaccharide found widely in cell walls of brown algae. It has been widely applied in food, cosmetic and pharmaceutical industries due to its unique physical properties to form gels (Wong et al. 2000). Alginate is composed of α-l-guluronic acid (G) and β-d-mannuronic acid (M) as structural units. Two kinds of hexuronic acid residues were linked by 1,4-*O*-glycoside bonds and can be degraded into alginate oligosaccharides (AOs) with low degree of polymerization by alginate lyases (Gacesa 1988; Ji 1997; Preiss and Ashwell 1962). As enzymatic degradation products of alginate, AOs are small and unsaturated alginate fragments with double bonds at the non-reducing end (Preiss and Ashwell 1962; Zhu and Yin 2015). These fragments with different structure and size exhibit various biological activities such as promotion of plant growth, anti-microbial, anti-oxidant, anti-tumor and immunomodulation (Falkeborg et al. 2014; Khan et al. 2012; Park et al. 2016; Saigusa et al. 2015; Wan et al. 1992; Xu et al. 2015; Yang et al. 2015; Zhou et al. 2015).

Alginate lyases are important and indispensable tools for production of AOs with special bioactivities (Wong et al. 2000; Zhu and Yin 2015). However, there are almost no commercialized alginate lyases with low cost and high yield until now. The narrow substrate specificity and low enzyme activity of existing enzymes still limit the industrial production of alginate oligosaccharides (Dou et al. 2013). Therefore, it is essential to continuously search and identify novel alginate lyases with high enzyme activity and wide substrate specificity from different sources. Nowadays, hundreds of alginate lyases have been isolated and identified from algae, marine mollusks and many kinds of microorganisms (Kawamoto et al. 2006; Matsubara et al. 2000; Zhu et al. 2015b, 2016a, b). Especially, different microorganisms have been reported to degrade alginate and become the most important sources of alginate lyases.

In order to acquire more alginate lyase-excreting microbes, a simple and efficient screening method needs to be developed. As an easy and convenient testing method, plate assay associated with constrain condition is traditionally used for bacteria screening. A number of alginate degrading strains were isolated by plate assay using different reagents as enzyme-producing indicator (e.g., cetyl pyridinium chloride, ruthenium red, calcium chloride, ethanol) (Gacesa and Wusteman 1990; Baron et al. 1994; Takeshita et al. 1991; Huang et al. 2013). Generally, a clear zone would occur around the alginate lyase-excreting colony after using the chromogenic agents. These qualitative methods make it possible to visualize the enzyme activity and can be applied to detect alginate lyase produced by bacterial colonies. However, the indistinct border and long reaction time of existing methods make the judgment of clear zones fairly difficult and inconvenient. The existing methods are still low efficient for revealing alginate lyase-excreting microbes at large scale. Recently, Sawant et al. (2015) reported that Gram’s iodine was an excellent chromogenic agent for revealing alginate lyase-producing colonies on agar plates. According Sawant’s result, Gram’s iodine could form distinct clear zones around the alginate degrading microbial colonies within 2–3 min. It was more effective than other chromogenic agents.

In this paper, we applied Gram’s iodine as chromogenic agent and a modified plate method with oxford cup to screening and compare alginate lyase-excreting microorganisms that are associated with the brown algae. Our goal is to screen novel alginate lyases at large-scales.

## Materials and methods

### Algae collection and bacteria isolation

Three kinds of brown algae *Laminaria japonica* (*L. japonica*), *Sargassum horneri* (*S. horneri*) and *Sargassum siliquatrum* (*S. siliquatrum*) were collected from the coast of Nanhuangcheng Island, China (38°21′N; 120°54′E), transferred to our laboratory and kept at 4 °C in a refrigerator. Each seaweed sample was cut into small pieces (2 cm × 2 cm). Ten grams of every sample were placed in a petri dish with 10 ml sterile water. The petri dishes were incubated at 30 °C until bacteria growing on the surface of seaweeds were easily observed with naked eyes (2–5 weeks). The bacteria were collected and diluted with sterile water. Aliquots (100 μl) of diluted sample were spread on screening plates consisted of 0.5% sodium alginate, 0.5% ammonium sulfate, 0.2% dipotassium phosphate, 0.1% magnesium sulfate, and 2% agar (ALG plates) (pH 7.2–7.4). The plates were incubated at 30 °C and checked daily until colonies were visible with naked eyes. Morphologically different colonies were numbered and transferred onto a new plate with the same medium. The new plate was incubated at 30 °C to form visible colonies, while the original plates were stained with Gram’s iodine solution (Sigma, St. Louis, USA, catalogue HT902). The clear zone around the strain colony indicated that the strain secreted alginate lyase into the medium (Sawant et al. 2015). The number of strains with clear zone was recorded and these strains were selected for further analysis.

### Amplification, sequencing and phylogenetic analysis of the 16S rDNA

The genomic DNA of each strain was extracted by the EasyPure genomic DNA kit (Transgen Biotech, Beijing, China, catalogue #EE101-01). The 16S rRNA gene was amplified by PCR using primers 27F (5′-AGAGTTTGATCCTGGCTCAG-3′) and 1492R (5′-TACGGTTACCTTGTTACGACTT-3′). The enzyme was EasyPfu DNA polymerase (Transgen Biotech, Beijing, China, catalogue #AP211-01). The amplified DNA fragments were sequenced by Sangon Biotech Inc. (Shanghai, China). The sequencing data were analyzed using Basic Local Alignment Search Tool (BLAST 2.3.0) (https://blast.ncbi.nlm.nih.gov/Blast.cgi) and bacterial species were identified based on sequence identity from the database. Phylogenetic trees were constructed using neighbor-joining method and Kimura two parameter model in MEGA 5.1 program (Kumar et al. 2008).

### Gram staining and microscopic observation

Fresh cells grown on ALG plate were picked up and spread on a microscope slide and stained with crystal violet (Beveridge 2001). The cells were observed with an optical microscope (Olympus BX51) using a 100× objective. Images were taken using an Olympus DP72 camera.

### Alginate lyase activity assay

The identified strains were inoculated into 100 ml of liquid ALG medium (pH 7.0) and incubated at 30 °C for 24 h with constant agitation (200 rpm). The culture was centrifuged at 12,000 rpm for 5 min and the supernatant were collected. Protein concentrations of the supernatants were detected using a protein quantitative kit (TransGen Biotech, Beijing, China).The enzyme activity was measured by two different methods:

#### Gram’s iodine method

200 μl of supernatant were added into an oxford cup and was placed on the surface of the ALG plate. The plates were incubated at 30 °C for 24 h and then stained with Gram’s iodine solution. The inner and outer diameters of the cleared zone were measured and the total area of the cleared zone was calculated. One unit of enzyme activity was defined as the amount of enzyme required to increase the cleared zone area by 0.1 mm^2^/min.

#### Ultraviolet absorption method

200 μl of supernatant were mixed with 1.8 ml liquid ALG medium. The mixtures were incubated at 30 °C for 24 h. The mixtures were heated to 100 °C for 10 min to stop the reaction. Absorbance at 235 nm was measured to determine the enzyme activity. One unit of enzyme activity was defined as the amount of enzyme required to increase 0.1 of the absorbance value at 235 nm/min (Preiss and Ashwell 1962).

### Determination of optimum conditions for producing alginate lyase

Effects of temperature, pH and alginate concentration on alginate lyase activity were tested using the selected enzyme-excreting strains. The strain incubated in 100 ml of ALG medium at 30 °C, 200 rpm for 24 h at various temperature, pH, time intervals and alginate sodium concentrations. The effect of temperature on alginate lyase activity was determined at temperature ranging from 20 to 40 °C. The pH effect was determined at pH ranging from 5 to 9 at an interval of 1-pH unit. The effect of alginate sodium concentrations was determined at the concentrations ranging from 0 to 1.2% at an interval of 0.2% unit. The effect of fermentation time was determined at time ranging from 0 to 24 h at an interval of 4 h. Unless otherwise specified, all fermentations were performed at 30 °C and pH 7.0. Alginate lyase activity was measured as described above.

### Detection of degradation product of alginate lyase

Thin layer chromatography (TLC) was applied to analyze alginate oligosaccharides produced by novel alginate lyase-exerting stains. TLC was performed according to the method described by Huang et al. (2013) and the loading volume was 2 μl. Fermentation samples at different time were collected and spotted on a TLC plate (Merck, Germany) with 1-butanol/formic acid/water (4:6:1 v:v:v) as a mobile solvent. Then the plate was sprayed with 10% (v/v) sulfuric acid in ethanol and heated at 110 °C for 10 min.

The fermentation broth of LJ-3 with AOs was collected and precipitated by adding ethanol (3 volumes) to remove higher molecular weight components. The solution was centrifuged at 12,000 rpm centrifuging for 15 min, then the supernatant was freeze-drying and then re-dissolved in deionized water. ESI–MS analysis was performed using a LCQ Fleet mass spectrometer (Thermo Fisher Scientific, USA) in negative mode.

### Sequences accession and strain deposition

16S rRNA gene sequences of identified alginate lyase-excreting strains were submitted and available in the GenBank Database under the Accession Number KX959962–KX959973. The deposition number of LJ-3 was CGMCC 12,155.

## Results

### Identification of alginate lyase-excreting bacteria

**Fig. 1:**
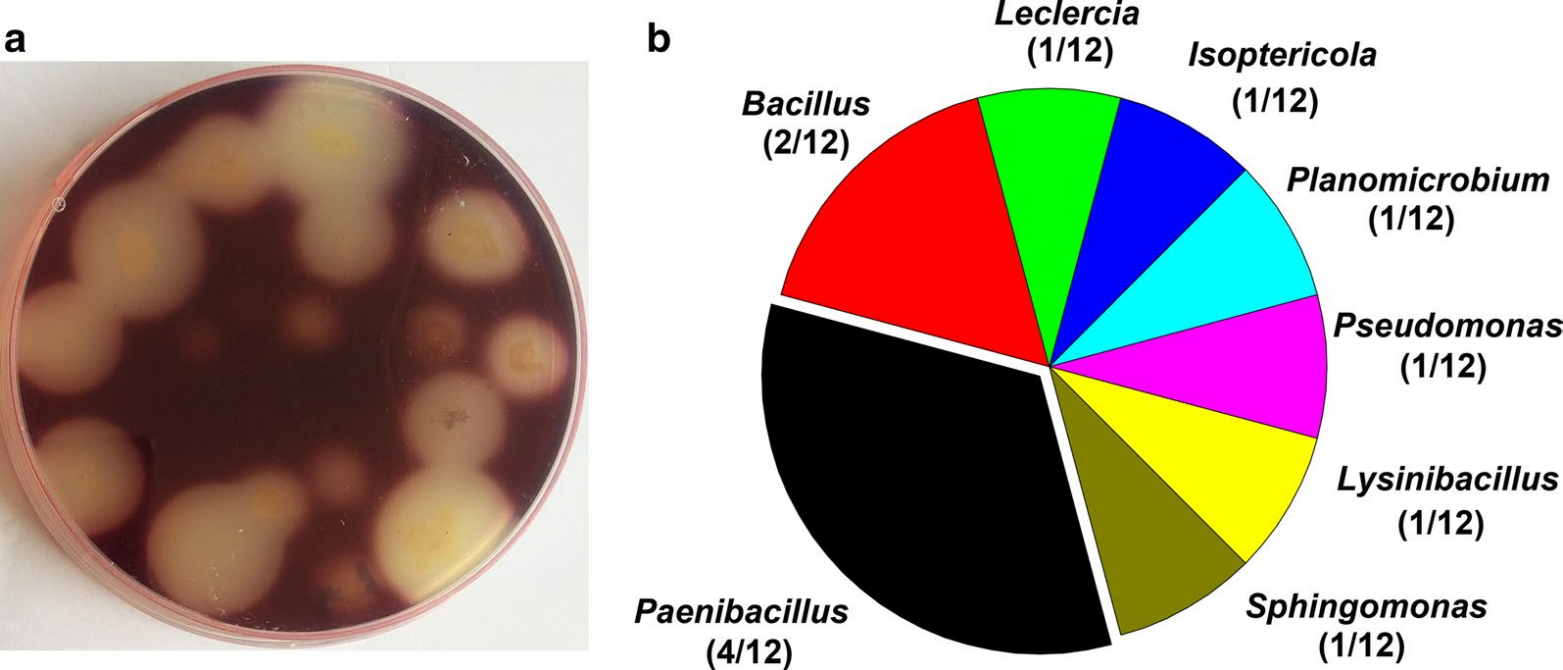
Isolation and identification of alginate lyase-excreting strains. **a** Gram’s iodine staining, showing the distinct zones of clearance of the alginate lyase-excreting strains (one of the screening plates for result exhibition). **b** Abundances of the alginate lyase-excreting bacteria strains

**Fig. 2:**
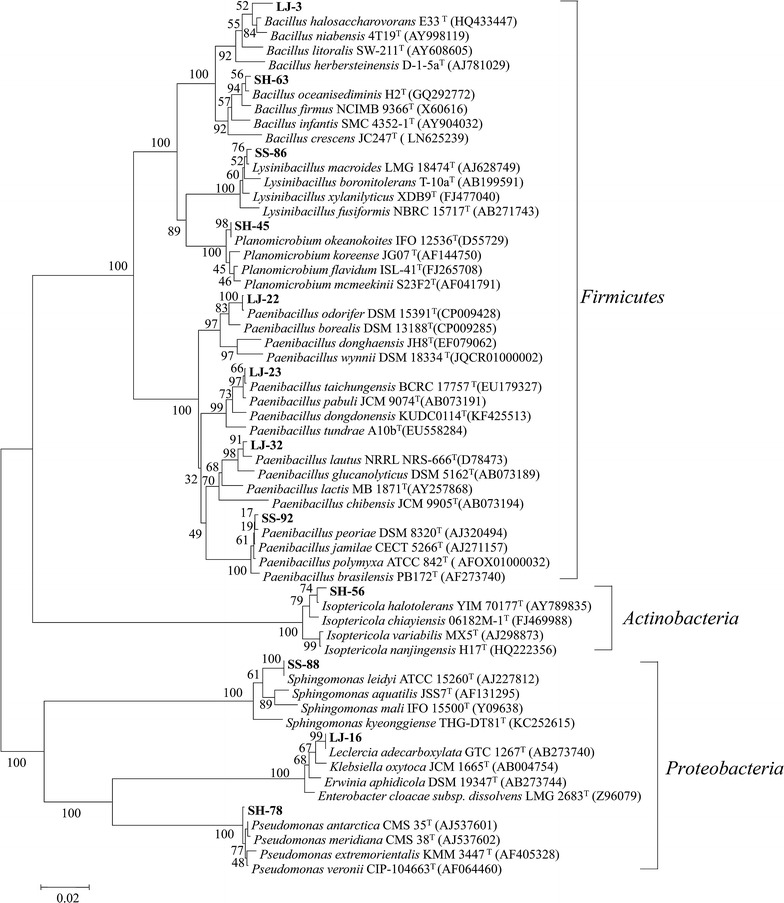
Neighbor-joining phylogenetic tree of the 12 alginate lyase-excreting bacteria based on the 16S rDNA sequences. Phylogenetic tree was constructed using neighbor-joining method with bootstrap (1000 replicates) by Kimura 2-parameter model using MEGA 5.1 program

Our primary screening identified 196 colonies from the three brown algae [106 from *L. japonica* (LJ), 53 from *S. horneri* (SH) and 37 from *S. siliquatrum* (SS)]. Gram’s iodine staining showed that 46 colonies had distinct zones of clearance (Fig. 1a). These 46 strains with clearance zone were selected and subjected to 16S rRNA gene amplification. Sequence analysis revealed that many of the 16S rRNA gene sequences were the identical, or only one base-pair difference and these are considered as the same bacterial strain. After removing the duplicates, a total of 12 different strains were identified, including 5 from *L. japonica*, 4 from *S. horneri* and 3 from *S. siliquatrum*. BLAST analysis revealed that bacterial strains belonged to eight genera, namely *Paenibacillus* (4/12), *Bacillus* (2/12), *Leclercia* (1/12), *Isoptericola* (1/12), *Planomicrobium* (1/12), *Pseudomonas* (1/12), *Lysinibacillus* (1/12) and *Sphingomonas* (1/12) (Table 1, Fig. 1b). Neighbor-joining phylogenetic analysis based on the 16S rRNA gene sequences further confirmed the identity of these 12 strains (Fig. 2). Gram staining and microscopic observation also showed that these strains were in accordance with their associated species (Table 1; Additional file 1: Figure S1).

**Table 1:** Identified isolates, each with the closest type strain, the 16S rRNA gene similarity and the observed enzyme activities

Strain ID	Closest bacterial strain (EZTaxon)	Identity (%)	Accession number	Gram staining	Diameter of clearance zone (cm)
LJ-3	*Bacillus halosaccharovorans*	98.07	KX959962	Positive	2.3 ± 0.2
LJ-16	*Leclercia adecarboxylata*	99.37	KX959963	Negative	0.8 ± 0.1
LJ-22	*Paenibacillus odorifer*	99.11	KX959964	Negative	1.4 ± 0.2
LJ-23	*Paenibacillus taichungensis*	99.25	KX959965	Negative	1.7 ± 0.2
LJ-32	*Paenibacillus lautus*	99.21	KX959966	Negative	1.7 ± 0.2
SH-45	*Planomicrobium okeanokoites*	99.24	KX959967	positive	1.2 ± 0.1
SH-56	*Isoptericola halotolerans*	98.69	KX959968	positive	1.6 ± 0.2
SH-63	*Bacillus oceanisediminis*	99.64	KX959969	positive	1.4 ± 0.1
SH-78	*Pseudomonas antarctica*	99.71	KX959970	Negative	1.1 ± 0.1
SS-86	*Lysinibacillus macroides*	99.26	KX959971	Positive	0.8 ± 0.1
SS-88	*Sphingomonas leidyi*	99.06	KX959972	Negative	1.1 ± 0.1
SS-92	*Paenibacillus jamilae*	99.27	KX959973	Negative	1.5 ± 0.2

### Evaluation of alginate lyase activities

**Fig. 3:**
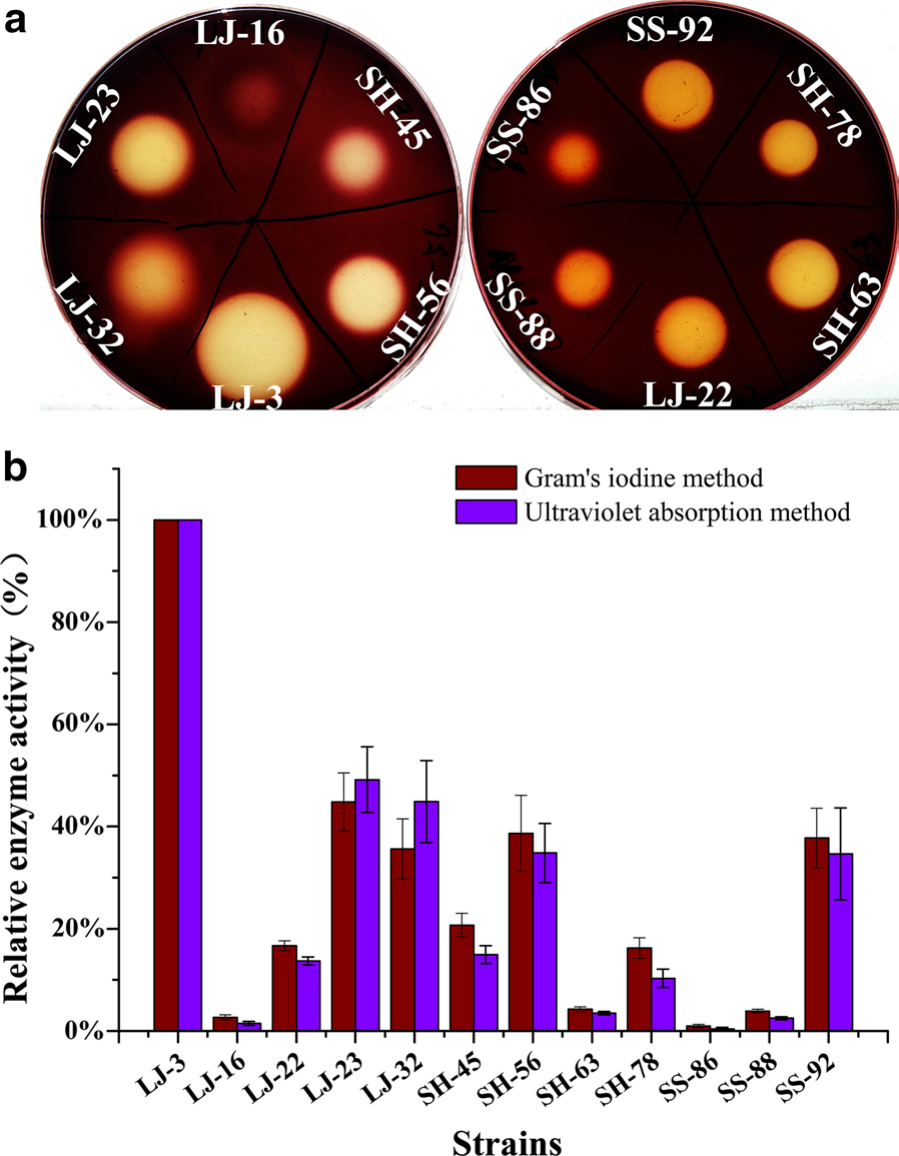
Quantification of alginate lyase activity. **a** Gram’s iodine method showing the clearance zone of twelve strains; **b** quantification of alginate lyase activities by Gram’s iodine method and ultraviolet absorption method. The enzyme activities of LJ-3 strain detected by both methods were taken as 100%, respectively. Each value represents the mean of three replicates ± standard deviation

As shown in Fig. 3a, the oxford cup assay showed that alginate lyase activity varies among the 12 different strains. LJ-3 strain (*Bacillus halosaccharovorans*) showed the highest alginate lyase activity, with a clearance zone diameter of 2.3 cm (Table 1). This result was further confirmed by the ultraviolet absorption method, which showed the LJ-3 had the highest enzyme activity, followed by LJ-23 (*Paenibacillus taichungensis*), LJ-32 (*Paenibacillus lautus*), SH-56 (*Isoptericola halotolerans*) and SS-92 (*Paenibacillus jamilae*) (Fig. 3). The ultraviolet absorption method was applied to verify the accuracy of Gram’s iodine method. As shown in Fig. 3b, the variation tendencies of enzyme activity measured by two methods were consistent.

### Optimization of growth conditions for production of alginate lyase secreted by LJ-3 strain

**Fig. 4:**
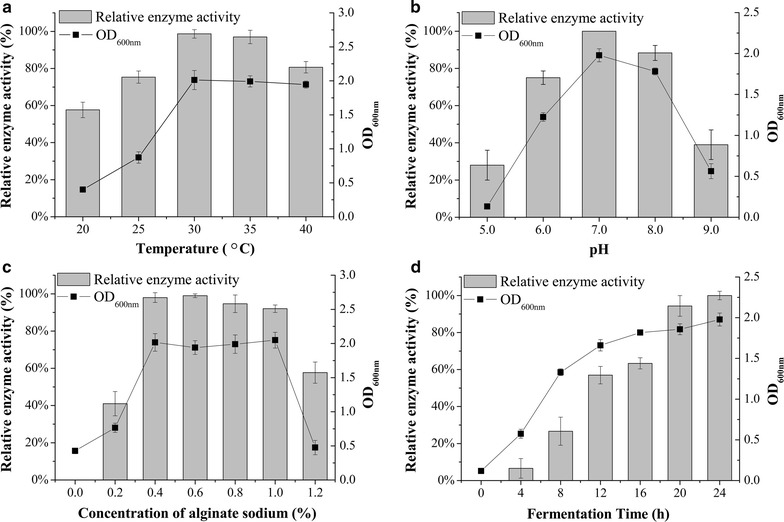
Optimization of growth conditions for production of alginate lyase secreted by LJ-3 strain. **a** Temperature; **b** pH; **c** sodium alginate concentration and **d** fermentation time. Each value represents the mean of three replicates ± standard deviation

The LJ-3 strain had the highest enzyme activity and therefore was chosen to perform further enzymatic at different temperature (Fig. 4a), pH (Fig. 4b), sodium alginate concentration (Fig. 4c) and fermentation time (Fig. 4d). Our results showed that the optimal condition for enzyme production was 30 °C, pH 7.0, 0.6% sodium alginate and 24 h. The relative enzyme activity was changed in accordance with the value of OD_600_. For example, the value of OD_600_ showed little change from 30 to 40 °C, so did the enzyme activity, which was maintained at level of above 80% (Fig. 4a). This result suggested that high microbial biomass produces a high yield of alginate lyase.

### Product analysis of alginate lyase-excreting strains

**Fig. 5:**
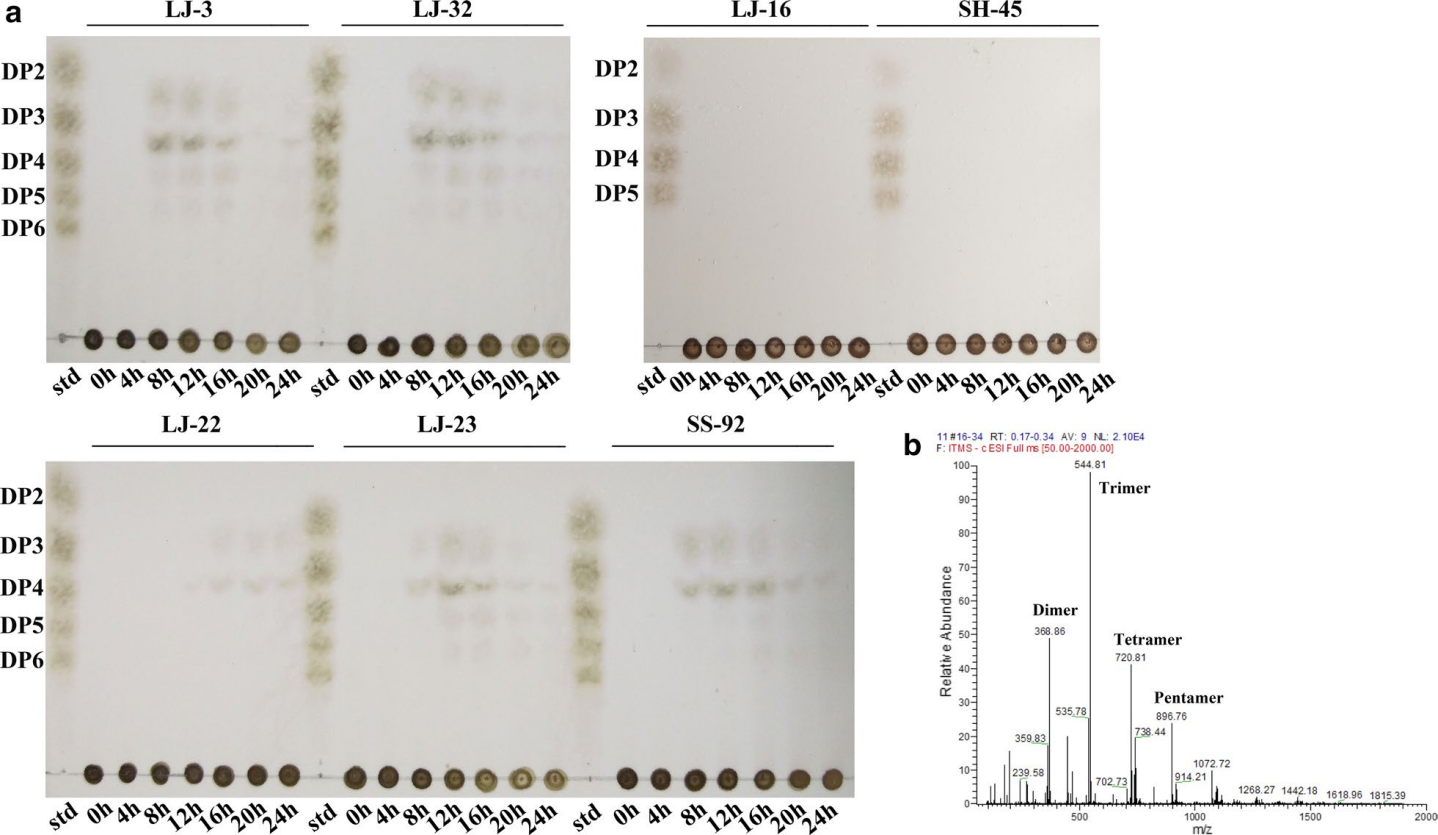
Thin layer chromatography (TLC) analysis of degrees of polymerization of the alginate oligosaccharides. Aliquot samples were taken at the interval of 4 h and then spotted on the TLC plate. *Std* alginate oligosaccharide standards. DP2–DP6 the mannuronic acid sodium salt dimer, trimer, tetramer, pentamer and heptamer

Thin layer chromatography (TLC) was applied to examine the alginate oligosaccharides (AOs) with different degrees of polymerization (DP) produced by different alginate lyase-excreting strains. The alginate lyase-excreting strains including four strains with high enzymatic activity (LJ-3, LJ-23, LJ-32 and SS-92) and three strains with relatively low enzymatic activity (LJ-16, LJ-22, SH-45) were selected for the tests. As shown in Fig. 5a, AOs were detected in majority of the fermentation broths except LJ-16 and SH-45. The degrees of polymerization of AOs and appearance time of AOs were different. Low molecular weight AOs (DP 2–5) were first detected at 8 h in fermentation broth of LJ-3 and LJ-32 strains. During 8–16 h, the amount of oligosaccharides gradually decreased. After 16 h, both DPs and the amount of oligosaccharides significantly decreased. The alginate oligosaccharides produced by LJ-3 strain were further confirmed by ESI–MS analysis. It was showed that the top 3 abundance oligomers were alginate oligosaccharide trimer, dimer and tetramer according to molecular weight determination (Fig. 5b). This result coincided with the TLC detection.

In fermentation broth of LJ-23 and SS-92 strains, AOs were also first detected at 8 h but the amount and DPs were maximized during 12–16 h. AOs were first detected at 16 h in fermentation broth of LJ-22 strain and only oligomers of DP2 and DP3 were detected. This result indicated that the alginate lyases of the five stains had different endolytic reaction mode. Thus, these alginate lyases could be useful tools for the preparation of alginate oligosaccharides with different DPs.

No AOs in fermentation broth of LJ-16 and SH-45 were detected. We inferred that the enzyme activities of LJ-16 and SH-45 strains were too low to produce enough oligosaccharides for detection by TLC or the consumption of oligosaccharides by strains was too fast to detect at 4-h sampling interval.

## Discussion

Bacteria screening is generally time consuming and labor-intensive. In our study, we applied Gram’s iodine method to isolate alginate lyase-excreting microorganism and confirmed that it was practical and convenient. The screening procedure was shown in Additional file 2: Figure S2. In combination with oxford cup method, we modified the Gram’s iodine method to achieve quantitative analysis of alginate lyase activity on a single plate. The quantification is validated with ultraviolet absorption method. The Gram’s iodine method requires no special equipment, making it suitable for large-scale screening and comparing of alginate lyase-excreting microorganism.

In the past several years, numerous alginate lyases have been isolated from different microorganisms, especially from those associated with brown algae. For example, 21 alginate lyase-excreting strains were isolated from the surface of the Arctic *Laminaria* and some cold-adapted alginate lyases were identified (Dong et al. 2012). More recently, Martin et al. (2015) isolated and identified 14 alginate lyase-excreting strains associated with the brown alga *Ascophyllum nodosum*, including one novel strain of *Marinomonas.* In this study, we identified 12 alginate lyase-excreting strains from the surfaces of three brown algae. Among them, novel alginolytic activity in *Paenibacillus*, *Leclercia* and *Planomicrobium* has not been previously reported. *Paenibacillus* produces many kinds of extracellular enzymes such as cellulose, proteases, amylase and other polysaccharide-degrading enzymes, which can be used in a wide range of industrial fields (Adlakha et al. 2015; Budi et al. 2000; Das et al. 2016; Dong et al. 2016; Lan Pham et al. 1998; Mathews et al. 2016). However, production of alginate lyase by *Paenibacillus* has never been reported. In this study, 4 alginate lyase-excreting strains belonging to *Paenibacillus* (LJ-22, LJ-23, LJ-32 and SS-92) were isolated from two different algae (*L. japonica* and *S. siliquatrum*). As shown in Additional file 3: Table S1, three of them (LJ-23, LJ-32 and SS-92) showed high enzyme activities compared to enzyme activities published previously (Li et al. 2015; Zhu et al. 2015a, b; 2016a). Our results indicated that *Paenibacillus* is a potential source for novel alginate lyase.

Although alginate lyases from the genus *Bacillus* were found in a few reports (Nakagawa et al. 1998), they may play an important role in aquaculture and agriculture. Recently, an alginate-degrading *Bacillus* strain was applied to improve the seaweed (*L. japonica*) based diet for sea cucumber (*Apostichopus japonicas*) (Wang et al. 2015). An alginate lyase-excreting strain (LJ-3) belonging to *Bacillus* genus was isolated in our study and it has potential to be used in industry due to its high enzyme activity. It could also be used as plant stimulant in agriculture. We showed that alginate oligosaccharides produced by LJ-3 strain could improve growth and quality of plants (unpublished data), especially promote early fruit coloring (Additional file 4: Figure S3).

## Additional files

**Additional file 1: Figure S1.** Gram staining of the 12 alginate lyase-excreting bacterial strains.

**Additional file 2: Figure S2.** Flow chart of screening procedure.

**Additional file 3: Table S1.** Comparison of alginate lyase activities in different strains.

**Additional file 4: Figure S3.** The fruit coloring promotion effect of alginate oligosaccharide on different plants.

## Abbreviations

OD: optical density
SD: standard deviation
AOs: alginate oligosaccharides
DP: degrees of polymerization
LJ: *Laminaria japonica*
SH: *Sargassum horneri*
SS: *Sargassum siliquatrum*
ALG: alginate
