# Supplementary figures and images for: Screening of alginate lyase-excreting microorganisms from the surface of brown algae
Source: AMB Express. 2017 Apr 4;7:74. doi: 10.1186/s13568-017-0361-x (PMC5378567; doi:10.1186/s13568-017-0361-x)

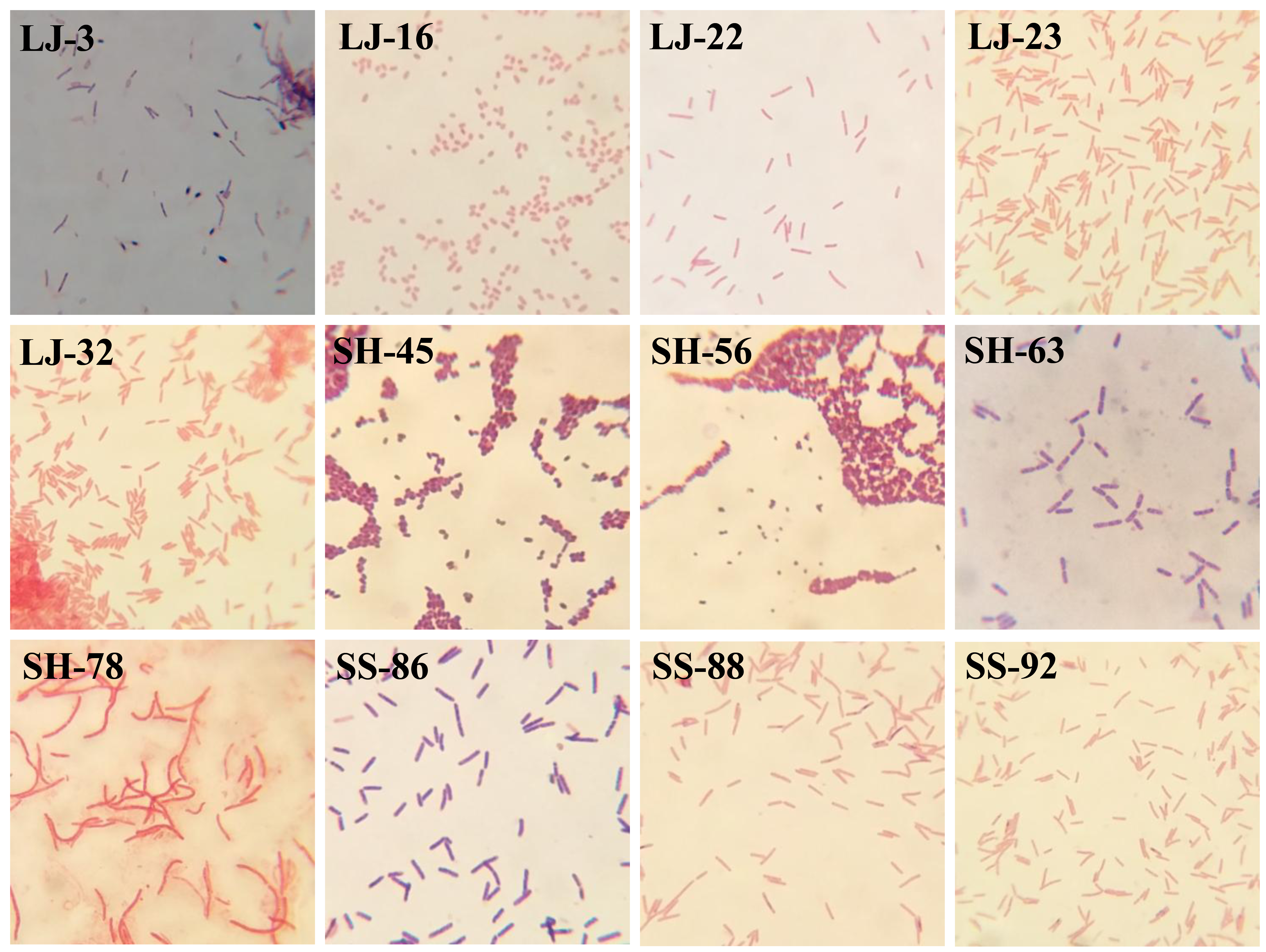

Supplement: Supplementary file 1 — Additional file 1: Figure S1. Gram staining of the 12 alginate lyase-excreting bacterial strains. [file 13568_2017_361_MOESM1_ESM.tif]

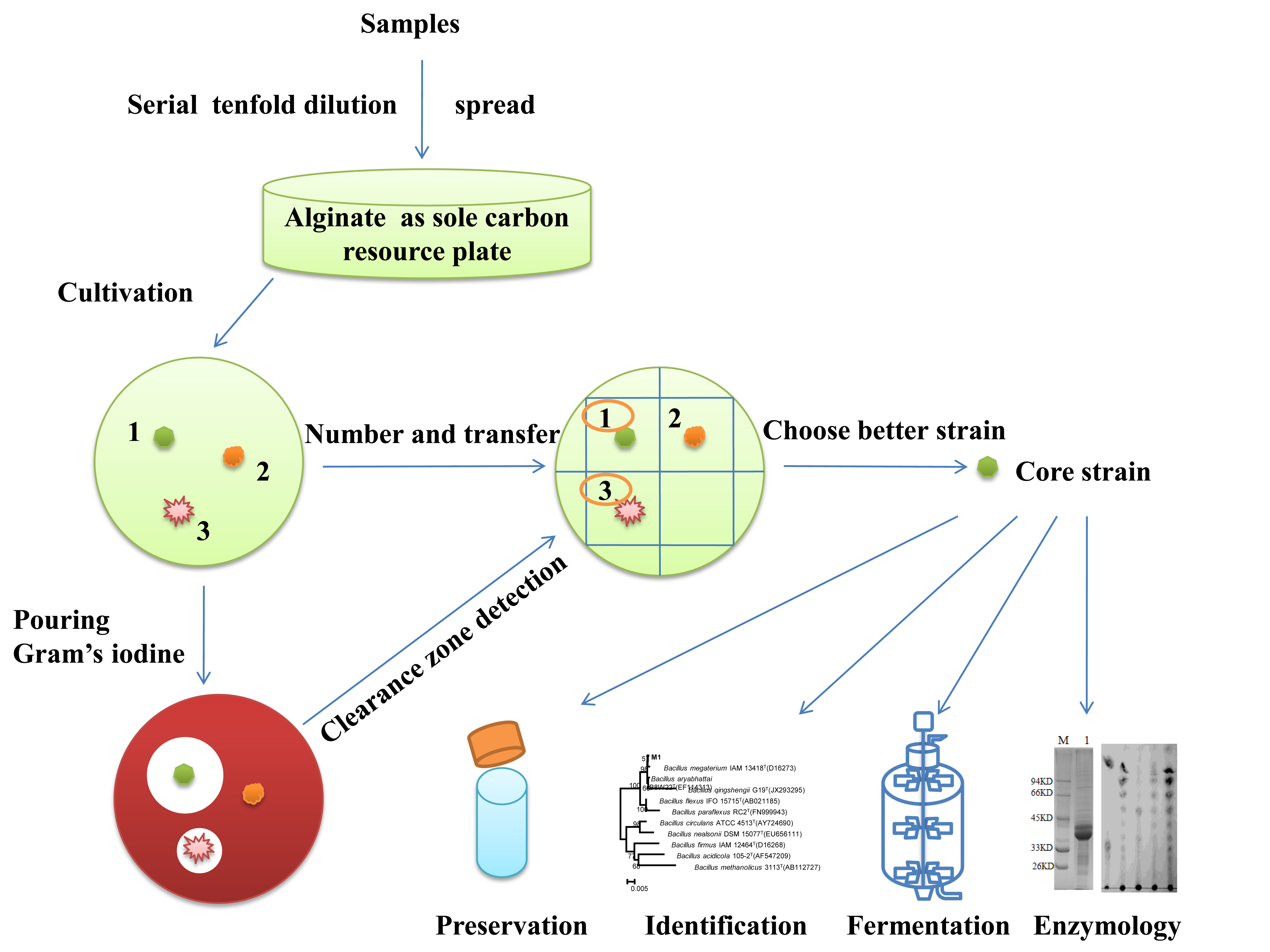

Supplement: Supplementary file 2 — Additional file 2: Figure S2. Flow chart of screening procedure. [file 13568_2017_361_MOESM2_ESM.tif]

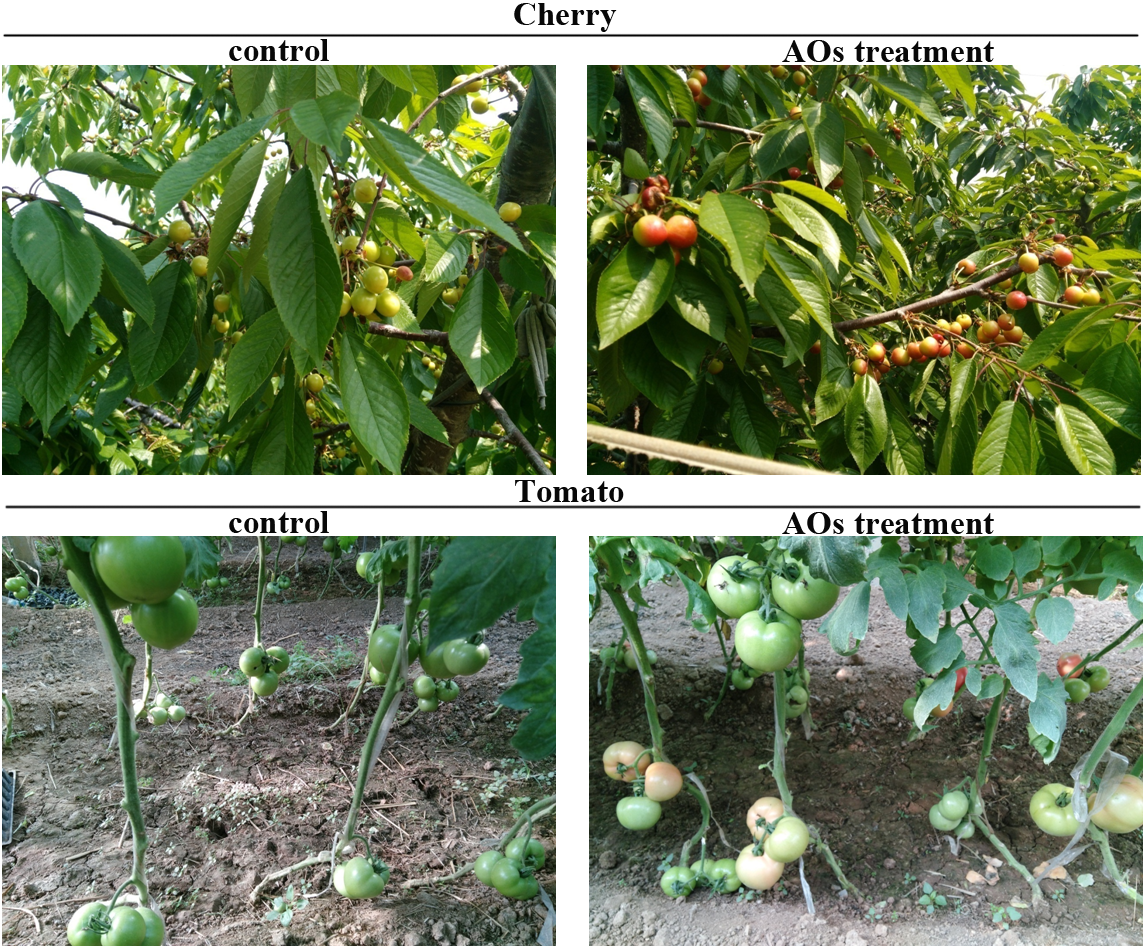

Supplement: Supplementary file 4 — Additional file 4: Figure S3. The fruit coloring promotion effect of alginate oligosaccharide on different plants. [file 13568_2017_361_MOESM4_ESM.tif]
