# Supplementary material for: Screening of alginate lyase-excreting microorganisms from the surface of brown algae
Source: AMB Express. 2017 Apr 4;7:74. doi: 10.1186/s13568-017-0361-x (PMC5378567; doi:10.1186/s13568-017-0361-x)
Supplement: Supplementary file 3 — Additional file 3: Table S1. Comparison of alginate lyase activities in different strains. [file 13568_2017_361_MOESM3_ESM.docx]

**Table S1 Comparison of alginate lyase activities in different strains**

| strains | species | Total protein(mg) | Total activity(U) | Specific activity*(U/mg) |
| --- | --- | --- | --- | --- |
| LJ-23 | *Paenibacillus* sp. | 2.24 | 24 | 10.71 |
| LJ-32 | *Paenibacillus* sp. | 2.82 | 27 | 9.57 |
| SS-92 | *Paenibacillus* sp. | 2.07 | 17 | 8.21 |
| NJ-01^a^ | *Cellulophaga* sp. | 1331 | 10055 | 7.55 |
| W13^b^ | *Vibrio* sp | 16.10 | 1220 | 75.77^Re^ |
| L11^c^ | *Agarivorans* sp. | - | - | 30.24 ^Re^ |

* One unit was defined as the amount of enzyme required to increase the absorbance at 235 nm by 0.1 per min.

a Data from Zhu et al. (2016a)

b Data from Zhu et al. (2015b)

c Data from Li et al. (2015)

Re, The recombinant alginate lyase expressed in *E. coli* BL21 (DE3).

-, not mentioned
